# Pacing across the membrane: the novel PACE family of efflux pumps is widespread in Gram-negative pathogens

**DOI:** 10.1016/j.resmic.2018.01.001

**Published:** 2018

**Authors:** Karl A. Hassan, Qi Liu, Liam D.H. Elbourne, Irshad Ahmad, David Sharples, Varsha Naidu, Chak Lam Chan, Liping Li, Steven P.D. Harborne, Alaska Pokhrel, Vincent L.G. Postis, Adrian Goldman, Peter J.F. Henderson, Ian T. Paulsen

**Affiliations:** a School of Environmental and Life Sciences, University of Newcastle, Callaghan, NSW, Australia; b School of BioMedical Sciences and Astbury Centre for Structural Molecular Biology, University of Leeds, Leeds, UK; c Department of Chemistry and Biomolecular Science, Macquarie University, North Ryde, NSW, Australia; d Biomedicine Research Group, Faculty of Health and Social Sciences, Leeds Beckett University, Leeds, UK; e Division of Biochemistry, Faculty of Biological and Environmental Sciences, University of Helsinki, Helsinki, Finland

**Keywords:** Membrane transport, Gram-negative pathogen, Antimicrobial resistance, Efflux, PACE, Bacterial transmembrane pair domain

## Abstract

The proteobacterial antimicrobial compound efflux (PACE) family of transport proteins was only recently described. PACE family transport proteins can confer resistance to a range of biocides used as disinfectants and antiseptics, and are encoded by many important Gram-negative human pathogens. However, we are only just beginning to appreciate the range of functions and the mechanism(s) of transport operating in these proteins. Genes encoding PACE family proteins are typically conserved in the core genomes of bacterial species rather than on recently acquired mobile genetic elements, suggesting that they confer important core functions in addition to biocide resistance. Three-dimensional structural information is not yet available for PACE family proteins. However, PACE proteins have several very highly conserved amino acid sequence motifs that are likely to be important for substrate transport. PACE proteins also display strong amino acid sequence conservation between their N— and C-terminal halves, suggesting that they evolved by duplication of an ancestral protein comprised of two transmembrane helices. In light of their drug resistance functions in Gram-negative pathogens, PACE proteins should be the subject of detailed future investigation.

## 1 Introduction

In the broadest sense, drug resistance may arise in actively growing bacterial cells in two distinct ways: either the drug target site is protected from the toxic activities of the drug by modification or bypass, or the drug cannot reach the target site due to degradation, sequestration, reduced cellular entry or active efflux. Efflux is a major mechanism of drug resistance, and due to the high promiscuity in substrate recognition by the transport proteins involved, efflux-mediated resistance is found for a wide range of different antimicrobial compounds.

Drug efflux proteins from five distinct families of transport proteins were described between the 1970s and 2000, and have been studied extensively at both the functional and structural levels [1]. These families include the ATP-binding cassette (ABC) superfamily, the major facilitator superfamily (MFS), the resistance-nodulation-cell division (RND) superfamily, the multidrug/oligosaccharidyl-lipid/polysaccharide (MOP) flippase superfamily and the drug/metabolite transporter (DMT) superfamily. In the last five years two new transporter families that include bacterial drug efflux systems have been identified; these are the proteobacterial antimicrobial compound efflux (PACE) family and the p-aminobenzoyl-glutamate transporter (AbgT) family [2], [3], [4]. Proteins from the PACE family transport biocides such as chlorhexidine and acriflavine, whereas AbgT family transporters transport sulphonamides.

## 2 The *Acinetobacter baumannii* AceI protein is a prototype for the novel PACE family of transport proteins

Drug efflux systems, and drug resistance factors in general, are frequently controlled by regulators that sense the transported drug substrates or their downstream effects in the cell. For example, TetR controls expression of the *tetB* tetracycline transporter gene in response to tetracyclines, and QacR controls expression of the multidrug efflux pump gene *qacA* in response to cationic antimicrobials [5]. For bacterial cells, this regulatory control means that efflux pump gene expression will proceed only when the pumps are required, saving cellular resources and preventing the potential toxic effects of constitutive high-level efflux pump expression [6]. From a research perspective, this tight regulatory control of drug efflux pump genes means that transcriptional changes may be used to identify either efflux pumps that might recognise substrates of interest, or novel factors that may be involved in drug resistance or tolerance.

The *Acinetobacter* chlorhexidine efflux protein (AceI) was identified by analysing the transcriptomic response of *A. baumannii* to the membrane active biocide chlorhexidine [2], [7]. *A. baumannii* has recently emerged as major cause of Gram-negative infections in hospitals. Strains of *A. baumannii* are becoming increasingly resistant to antibiotics. Consequently, carbapenem-resistant strains of *A. baumannii* are listed as “Priority 1: CRITICAL” targets for development of new antibiotics World Health Organisation [8]. Chlorhexidine is listed as an essential medicine by the World Health Organisation, and is commonly used as an antiseptic in wound dressings, hand washes and mouthwashes. The transcriptome of *A. baumannii* ATCC 17978 cells exposed to a subinhibitory concentration of chlorhexidine, equivalent to half the minimum inhibitory concentration, was compared to control cells. The major gene expression changes were to genes encoding the AdeAB components of the AdeABC multidrug efflux pump and a gene annotated as encoding a hypothetical protein, A1S_2063 [2].

**Fig. 1:**
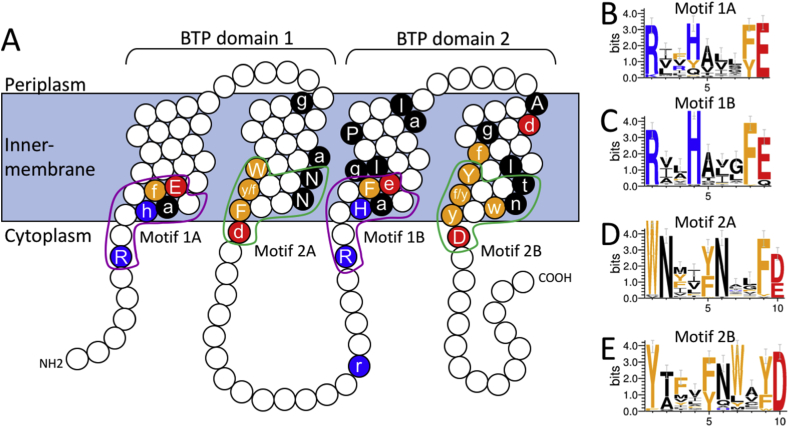
Predicted transmembrane topology and conserved amino acid sequence motifs present in PACE family proteins. An amino acid sequence alignment of 47 diverse PACE family proteins (Supplemental Fig. S1), encoded by a broad range of hosts, was used to identify amino acid sequence motifs that are conserved across the family (A) Predicted topology of PACE family proteins. Upper case characters are conserved in greater than 90% of protein sequences and characters in lower case are conserved in greater than 65% but fewer than 90% of the aligned protein sequences. Conserved residues are coloured (red: negatively charged, blue: positively charged, orange: aromatic, black: others). The similar amino acid sequence motifs in helices 1 and 3 (1A and 1B, respectively) are surrounded by purple lines, and the similar sequence motifs in helices 2 and 4 (2A and 2B, respectively) are surrounded by green lines (B–E) Sequence logos, made using WebLogo 3 [24], showing conservation of amino acid residues in the four sequence motifs identified in PACE proteins; error bars show an approximate Bayesian 95% confidence interval [24]. (For interpretation of the references to color/colour in this figure legend, the reader is referred to the Web version of this article.)

From its sequence, the A1S_2063 gene was predicted to encode an inner membrane protein with four transmembrane helices (Fig. 1A). The gene was cloned into an *Escherichia coli* expression vector and was shown to confer significant levels of resistance to chlorhexidine when overexpressed in *E. coli*. Deletion of the A1S_2063 gene in *A. baumannii* ATCC 17978 and its orthologue in *Acinetobacter baylyi* ADP1 halved the chlorhexidine resistance in the host strain, demonstrating that the genes had a resistance function in native hosts [2], [9]. The Biolog Phenotype Microarray system was used to screen over 200 additional antimicrobials against A1S_2063 expressing *E. coli*. This analysis demonstrated no significant hits apart from chlorhexidine, suggesting that chlorhexidine had an apparent specificity as a substrate [2].

When overexpressed in *E. coli*, the A1S_2063 protein was identified in the inner membrane. The protein could be readily extracted from the membrane by detergent solubilisation and purified. The detergent-solubilised protein bound to chlorhexidine with high affinity (*K*_d_ in the low μM range) as determined by tryptophan fluorescence quenching and near-UV synchrotron radiation circular dichroism [2]. Transport experiments using [^14^C]-chlorhexidine demonstrated that the A1S_2063 protein prevented the high-level accumulation of chlorhexidine when expressed in *E. coli* until the proton-motive-force across the membrane was collapsed using a protonophore. The protein could also mediate the efflux of chlorhexidine from *E. coli* cells preloaded with chlorhexidine [2]. Together, these results suggested that the A1S_2063 protein was a novel chlorhexidine efflux protein and that transport is likely to be proton-coupled. The protein was named AceI (*Acinetobacter* chlorhexidine efflux protein I).

## 3 PACE proteins are a family of multidrug efflux systems conserved across many Gram-negative pathogens

Genes encoding proteins homologous to AceI are found in the genomes of many bacterial species, including pathogens such as *Pseudomonas*, *Klebsiella*, *Enterobacter*, *Salmonella* and *Burkholderia* species. These genes are particularly common among Proteobacteria, but can be found in some Actinobacteria and in a limited number of other unrelated bacterial species. To determine whether, like AceI, these proteins can mediate chlorhexidine resistance, more than 20 phylogenetically diverse homologues were cloned into an *E. coli* expression system and examined by routine minimum inhibitory concentration analyses. Most of the cloned proteins were expressed at detectable levels, and about half could confer resistance to chlorhexidine [3]. Notably, at least two of the *aceI* homologues found to confer chlorhexidine resistance are also highly expressed in their native hosts, *Pseudomonas aeruginosa* and *Burkholderia cenocepacia*, in response to a chlorhexidine treatment [10], [11].

Additional resistance tests were performed to determine whether the antimicrobial recognition profiles of these homologues might extend beyond chlorhexidine. Many of the proteins were able to confer resistance to several additional biocides, including acriflavine, proflavine, benzalkonium and dequalinium [3]. The substrate profile of one pump, VP1155 encoded by *Vibrio parahaemolyticus*, was investigated using the Biolog phenotype microarray system. In addition to chlorhexidine, benzalkonium, proflavine and acriflavine, VP1155 appeared to confer resistance to 9-aminoacridine, domiphen bromide, guanazole and plumbagin [3].

**Fig. 2:**
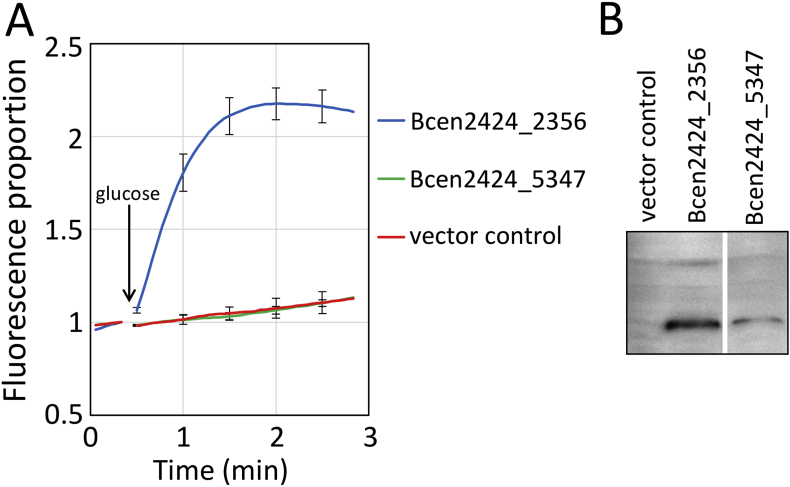
Acriflavine transport mediated by PACE family proteins encoded by the human pathogen *Burkholderia cenocepacia* HI2424. A) Transport experiments performed using *E. coli* OmniMax cells expressing the proteins of interest, essentially as described previously [3]. The cells were preloaded with 5 μM acriflavine in the presence of 10 μM CCCP. The cells were washed and re-energised using glucose at the point marked with an arrow and transport monitored fluorimetrically. Acriflavine fluorescence is quenched when it is intercalated into nucleic acids. Therefore, its transport out of the cell is associated with an increase in fluorescence. B) Western blot performed on the cells used in the assay, probed using His-Probe-HRP. The *B. cenocepacia* HI2424 PACE family proteins Bcen2424_2356 and Bcen2424_5347 were expressed at easily detectable levels after the 1-h induction used for this assay. Bcen2424_2356 promoted the rapid efflux of acriflavine, whereas Bcen2424_5347 did not promote acriflavine transport above background. The third PACE family protein encoded by *B. cenocepacia* HI2424, Bcen2424_5167 was not expressed and was excluded from the figure. Bcen2424_5167 and Bcen2424_5347 are phylogenetically distinct from Bcen2424_2356. Error bars show the standard deviation of three replicate experiments.

The demonstration that many AceI homologues are able to confer resistance to compounds such as proflavine and acriflavine presented the possibility of assaying transport by measuring their fluorescence in real time [12]. These compounds intercalate into nucleic acids, which leads to a quenching of their fluorescence. This property facilitates a convenient assay for their transport in cells expressing an efflux pump [13]. Cells expressing the protein of interest can be loaded with proflavine or acriflavine in the presence of a protonophore, such as carbonyl cyanide *m*-chlorophenylhydrazone (CCCP), then washed and re-energised by the addition of an energy source such as d-glucose. Fluorescence can be monitored before and after energisation to examine transport [12]. These transport experiments have been performed for a number of AceI homologues and identified proteins that mediate transport of these compounds. For example, the *B. cenocepacia* HI2424 homologue Bcen2424_2356 is able to transport acriflavine, whereas at least one other homologue encoded by this strain, Bcen2424_5347, does not (Fig. 2). Bcen2424_2356 has been previously shown to confer resistance to chlorhexidine, benzalkonium, proflavine and acriflavine. The Biolog phenotype microarray antimicrobial resistance tests confirmed several of these phenotypes and suggested that Bcen2424_2356 also confers resistance to benzethonium, 9-aminoacridine, methyl viologen, guanazole and plumbagin (Supplemental Fig. S2).

The observation that several AceI homologues can confer resistance to multiple biocides and can mediate transport of the fluorescent substrates proflavine and acriflavine led to their designation as a new family of efflux pumps. This family was called the Proteobacterial Antimicrobial Compound Efflux (PACE) family, due to their abundance in Proteobacteria [3]. To date at least ten putative substrates have been identified for PACE family proteins (Fig. S3). Proteins from this family have been incorporated into the Transporter Classification Database [14] under the original family title, the proteobacterial chlorhexidine efflux (CHX) family (TCDB number: 2.A.117), and are captured in the TransportDB 2.0 database [15], which catalogues all putative transport proteins from sequenced genomes in the NCBI RefSeq database.

## 4 Predicted topology and sequence conservation in PACE pumps

All PACE family proteins analysed to date are predicted to contain four transmembrane α-helices, organised into two tandem bacterial transmembrane pair (BTP) domains (Fig. 1; pfam: PF05232) [16]. Given their small size, it seems very likely that PACE proteins function as oligomers. However, the oligomeric state of PACE family proteins remains unresolved. Several PACE family proteins have been experimentally characterised by overexpression and purification (Henderson et al., unpublished). When expressed in *E. coli*, these proteins localise to the inner membrane and can be readily purified by extraction with a mild detergent such as n-dodecyl-β-D-maltoside [2], or using a styrene maleic acid co-polymer (Supplemental Fig. S4) [17]. Analysis of the purified detergent-solubilised proteins by far-UV circular dichroism has confirmed their high α-helical content and demonstrated that they typically show structural stability to around 50–60 °C [2].

A high level of amino acid sequence conservation is apparent between members of the PACE family. Based on an alignment of 47 diverse PACE family proteins from a variety of bacterial genera (Supplemental Fig. S1), four amino acid residues appear to be universally conserved across these proteins: a glutamic acid residue within transmembrane helix 1, an asparagine residue in transmembrane helix 2, an alanine residue at the periplasmic membrane boundary of transmembrane helix 4 and an aspartic acid residue at the cytoplasmic membrane boundary of transmembrane helix 4 (Fig. 1A). The functional importance of the conserved asparagine, alanine and aspartic acid residues has not yet been investigated, but neutralisation of the glutamic acid residue in the prototypical PACE family member AceI by substitution with a glutamine abolished chlorhexidine resistance and transport [2]. This mutant (E15Q) was still able to bind chlorhexidine with only slightly reduced affinity compared to the parental protein. Furthermore, the mutant protein was less thermostable than the parental protein in the absence of chlorhexidine, but was significantly more stable than the parental protein in the presence of a molar excess of chlorhexidine, supporting a binding interaction. These results suggest that the glutamic acid residue is not required for substrate binding. The high conservation of this residue in PACE proteins that have different substrate specificities suggests that it may be involved in an aspect of transport common to these proteins, such as an energy coupling reaction. The position of this residue within the transmembrane region is consistent with this possibility. In other efflux pumps from the MFS, SMR and MATE families, membrane embedded carboxyl residues participate in coupling reactions [18], [19], [20].

PACE family proteins contain several highly conserved amino acid residues in addition to the four universally conserved residues. The amino acid sequence conservation is particularly strong close to the predicted cytoplasmic boundaries of the transmembrane helices, where four amino acid sequence motifs have been identified (Fig. 1). In line with the PACE proteins containing tandem BTP domains, the amino acid sequence motif in transmembrane helix 1 (motif 1A; RxxhaxxfE, where upper case residues are conserved in more than 90% of proteins and lower case residues in at least 65% of proteins) is very similar to that in transmembrane helix 3 (motif 1B; RxxHaxxFe) (Fig. 1B and C), and the motif in transmembrane helix 2 (motif 2A, WNxxy/fNxxFd) is very similar to that in transmembrane helix 4 (motif 2B; Ytxxf/ynwxyD) (Fig. 1D and E). The notable features of the sequence motifs in helices 1 and 3 are the membrane-embedded glutamate residue (universally conserved in helix 1), and histidine and arginine residues at the membrane boundary. The motifs found in helices 2 and 4 notably contain several aromatic residues along one helical face adjacent to polar asparagine residues, and an aspartate residue at the membrane boundary (universally conserved in helix 4) (Fig. 1A).

Based on the distribution of charged residues within the loop regions, the N— and C-termini of most PACE family proteins are predicted to lie within the cytoplasm, but this is yet to be experimentally tested (Fig. 1). Some PACE family homologues, primarily from *Acetobacter*, contain predicted N-terminal signal sequences, suggesting that the N-terminus is moved across the cytoplasmic membrane and that they may exist in an alternative topology, e.g. APA01_04520 and APO_1949 from *Acinetobacter pasteurianus* IFO 3283-01 and *Acinetobacter pomorum* DM001, respectively. Representatives of these proteins have been expressed in *E. coli*, but as yet, no resistance or transport functions have been identified (Hassan et al., unpublished). These proteins may be part of a separate protein subfamily from those that mediate drug resistance.

## 5 Conservation of PACE family genes

PACE family proteins are typically highly conserved in the genome of the bacterial species in which they are found. For example, genes encoding three different PACE proteins have been identified in the *A. baumannii* pan-genome (based on the genomes of 623 strains) [7]. Of these, two were conserved in 100% or close to 100% of the strains and can be considered to be part of the core genome. The third gene was found in only two strains and is part of the accessory genome. Similar to *A. baumannii*, *P. aeruginosa* isolates have two PACE proteins encoded in the core genome and one in the accessory genome, which is found in only a few strains, and *B. cenocepacia* strains encode three PACE pumps in their core genome [7]. This high level of conservation suggests that PACE pumps are acquired vertically and have been maintained in their host species since their divergence from related organisms. They are thus likely to have an important core function that may be unrelated to drug resistance. Indeed, the biocides that are recognised by PACE family pumps have only been present in the environment for 50–100 years, and are therefore very unlikely to be the physiological substrates of these proteins.

In contrast to the species described above, *E. coli* do not encode PACE pumps in their core genomes; four different genes encoding PACE homologues were found among the genomes of 1986 sequenced *E. coli* strains, but these were each found in 0.2% of strains or less [7]. These accessory genes are likely to move between related species on mobile genetic elements. However, there is as yet no strong evidence for how these genes are mobilised.

## 6 Evolution of the PACE family

The conservation of sequence motifs between the N— and C-terminal halves of PACE proteins suggests that these proteins may have evolved by a duplication event of an ancestral single BTP domain protein. To investigate this further, the N— and C-terminal BTP domains were compared between 47 diverse PACE family proteins (Supplemental Fig. S5). The level of amino acid identity between the N— and C-terminal BTP domains in these proteins ranged from 6.6% to 33.3% (mean 23.3%). The presence of such high levels of sequence identity between the N— and C-terminal BTP domains across diverse PACE family proteins suggests that these proteins may not have diverged significantly since the occurrence of the duplication event(s). Along with the distribution of these proteins almost exclusively within the Proteobacteria, and their likely vertical acquisition, due to their presence on the core genome, this may suggest that this protein family is relatively young compared to other families of transport proteins, which show lower levels of sequence conservation between domains that are thought to have arisen via duplication [21].

To further examine the evolution of PACE family proteins, the levels of sequence identity between the N-terminal and C-terminal BTP domains of different PACE proteins were determined. It was found that the N-terminal BTP domains of PACE family proteins almost always have higher identity to the N-terminal BTP domains of other PACE proteins than they do to their own C-terminal BTP domain, or the C-terminal BTP domain of other PACE family pumps (Supplemental Fig. S5). This suggests that a BTP domain duplication event occurred only once in an ancestral gene, and that there is little or no recombination between the N— and C-terminal BTP domains in individual strains. The C-terminal BTP domains of different PACE pumps typically show even higher levels of sequence identity than the N-terminal domains (Supplemental Fig. S5). The high conservation of sequence within the C-terminal domain of different proteins may reflect the involvement of the C-terminal domain in a core part of the functional mechanism, whereas the N-terminal domain may play a bigger role in substrate recognition.

## 7 Concluding remarks

The PACE family of transport proteins is one of two transporter families discovered only recently to mediate drug efflux. From currently available analyses, PACE family proteins display somewhat restricted drug substrate recognition profiles, which include primarily synthetic biocides such as chlorhexidine and acriflavine, rather than the multitudes of diverse antibiotics and biocides recognised by transport proteins from families such as the RND superfamily. This may be a primary reason for the family being only recently identified, 15 years after the first descriptions of MATE family pumps [22], [23]. However, PACE proteins are highly conserved in a range of opportunistic Gram-negative pathogens, including *A. baumannii*, *P. aeruginosa*, *B. cenocepacia* and *Klebsiella pneumoniae*, and in serious human pathogens such as *Yersinia pestis*, *Francisella tularensis* and *Burkholderia pseudomallei.* Therefore, the role of these proteins in drug resistance warrants future investigation.

As mentioned above, the drug recognition profile of PACE pumps primarily includes synthetic biocides, most of which have only been in the environment for 50–100 years. However, genes encoding homologous PACE family proteins are found in the core genomes of bacterial genera that diverged much earlier than this, hundreds of millions of years ago. Therefore, these proteins are likely to mediate an important core function and may have common physiological substrates that are yet to be described. The importance of PACE family proteins is likely to extend beyond an apparently fortuitous role in drug resistance.

## Conflict of interest

The authors declare no conflict of interest.

## Figures and Tables

**Fig. s1:**
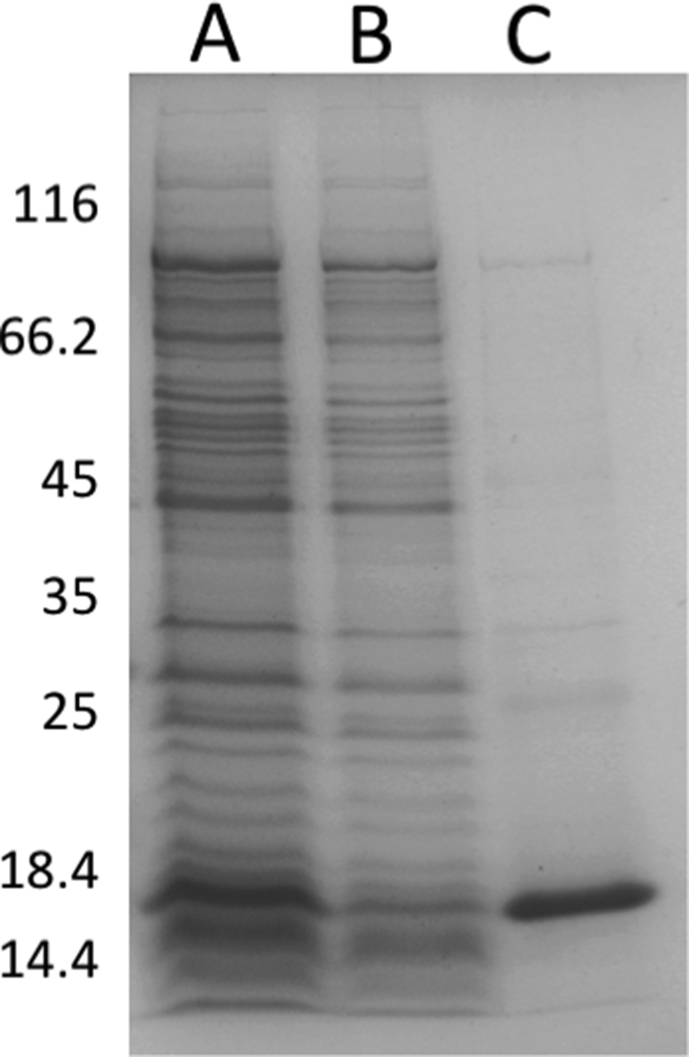
Solubilisation and purification of the *Ferrimonas balearica* DSM 9799 PACE family protein, Fbal_3166 [3], using styrene maleic acid co-polymer and Ni-affinity purification. Fbal_3166 protein was overexpressed in *E. coli* BL21 cells grown in a 30 L fermenter using the pTTQ18 expression system [27], [28]. Styrene maleic acid co-polymer preparation, membrane solubilisation and Ni-affinity purification were performed as previously described [17]. Samples consisting of solubilised membrane proteins (lane A), proteins that did not bind to the Ni-affinity column (lane B) and purified Fbal_3166 (lane C) were run on a 15% SDS-PAGE gel and stained with Coomassie brilliant blue R-250. The size (kDa) of co-migrated soluble molecular weight markers is shown on the left side of the gel.

## Appendix A Supplementary data

mmc1 Amino acid sequence alignment of 47 diverse PACE family proteins. The sequences are named by locus tag or NCBI protein accession. Sequences were obtained from the NCBI genomes database and aligned using ClustalX [25]. The alignment is coloured according to the level of amino acid sequence conservation at each position. Colours were added using the UGENE toolkit [26].

mmc2 Kinetic response curves parallelling bacterial growth for Biolog phenotype microarray antimicrobial tests in which the pTTQ18-Bcen2424_2356 plasmid facilitated a growth advantage. Curves for *E. coli* BL21 cells carrying pTTQ18 are shown in red, curves for BL21 cells carrying pTTQ18-Bcen2424_2356 are shown in green and regions of overlap in the response curves of these two strains are shown in yellow. The tests were conducted according to the manufacturer's instructions in the presence of 0.05 mM IPTG to promote expression of the cloned Bcen2424_2356 gene. The curves depict the colour intensity of a redox-active dye (y axis) over time (x axis; 30 h). (A) plate PM12, well E12 (benzethonium chloride) (B) plate PM14, well A3 (acriflavine), (C) plate PM14, well B3 (9-aminoacridine), (D) plate PM15, well E11 (methyl viologen), (E) plate PM18, well G7 (3,5-diamino-1,2,4-triazole [guanazole]), (F) plate PM18, well H12 (plumbagin), (G) plate PM19, well C4 (chlorhexidine).

mmc3 Putative substrates of PACE family proteins identified using whole cell transport experiments (chlorhexidine, acriflavine), conventional minimum inhibitory concentration analyses, (chlorhexidine, acriflavine, benzalkonium, dequalinium) and/or higher throughput resistance tests using the Biolog Phenotype Microarray system (chlorhexidine, acriflavine, benzethonium, 9-aminoacridine, methyl viologen, guanazole, plumbagin, domiphen). The chemical structures were obtained from the NCBI PubChem database and viewed using the web-based MolView tool (molview.org).

mmc4 Pairwise comparisons of the individual BTP domains in 47 diverse PACE family proteins. The amino acid sequences of the separated domains were analysed using the MatGat tool [29] to determine the percent identity between each domain. The amino acid sequence identities of each pair of domains are shown in the pairwise distribution plot and coloured according to percent identity (red: low identity, yellow: intermediate identity, and green: high identity). The top left region of the plot shows comparisons between the N-terminal BTP domains of different proteins and the bottom right region of the plot shows comparisons between the C-terminal BTP domains of different proteins. The central boxed region of the plot represents comparisons between the N-terminal domains and C-terminal domains of the proteins. The small boxes within this region highlight comparisons between the N— and C-terminal domains of the same protein.
