# Supplementary figures and images for: Pacing across the membrane: the novel PACE family of efflux pumps is widespread in Gram-negative pathogens
Source: Res Microbiol. 2018 Sep-Oct;169(7-8):450–4. doi: 10.1016/j.resmic.2018.01.001 (PMC6195760; doi:10.1016/j.resmic.2018.01.001)

Consensus

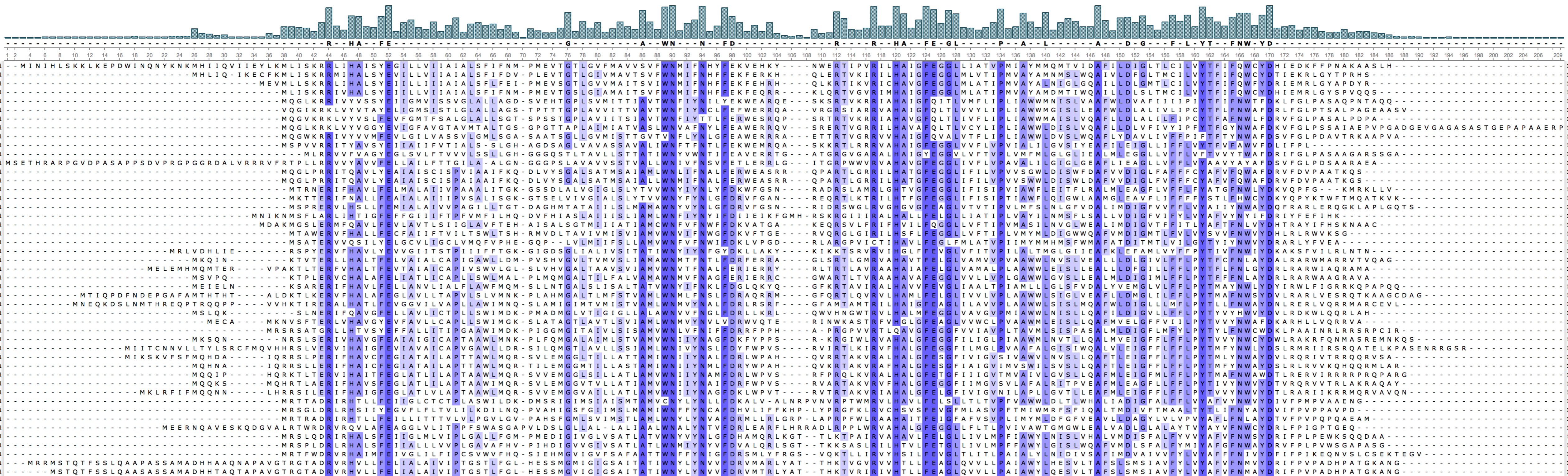

Supplement: mmc1 — Amino acid sequence alignment of 47 diverse PACE family proteins. The sequences are named by locus tag or NCBI protein accession. Sequences were obtained from the NCBI genomes database and aligned using ClustalX [25]. The alignment is coloured according to the level of amino acid sequence conservation at each position. Colours were added using the UGENE toolkit [26]. [file mmc1.pdf]

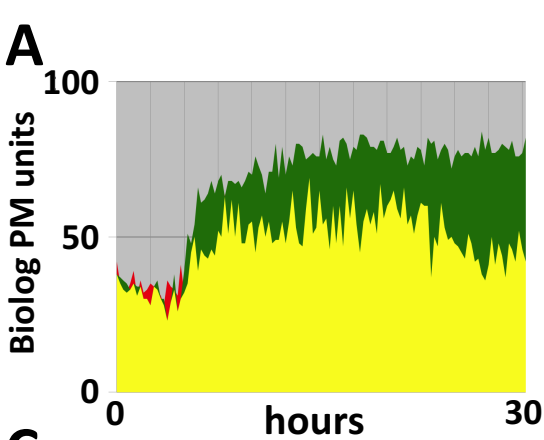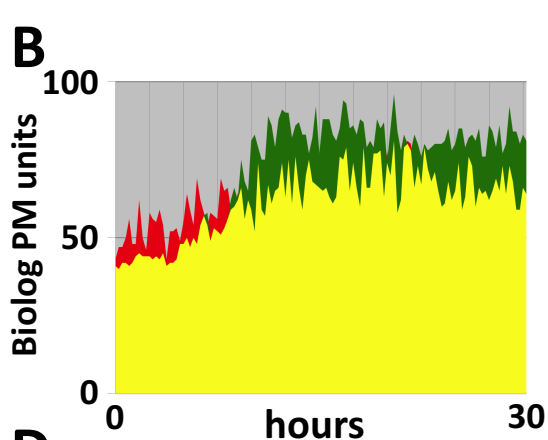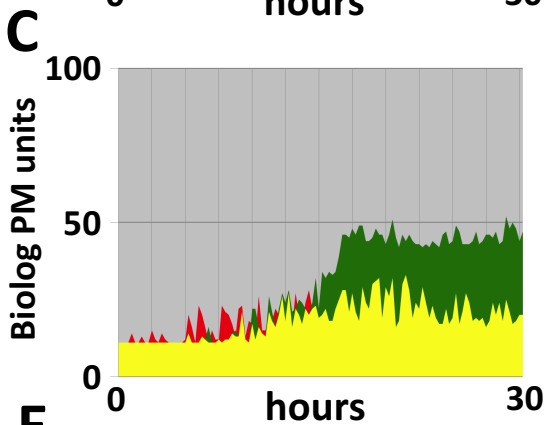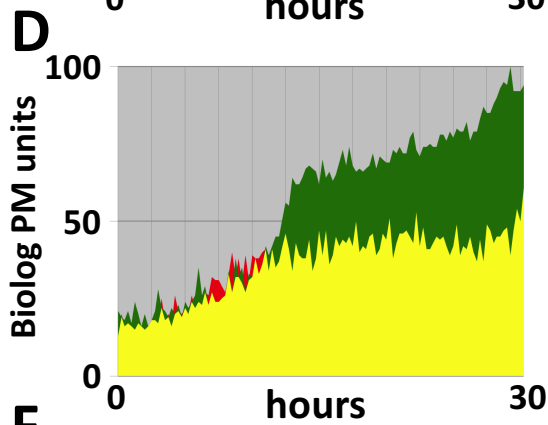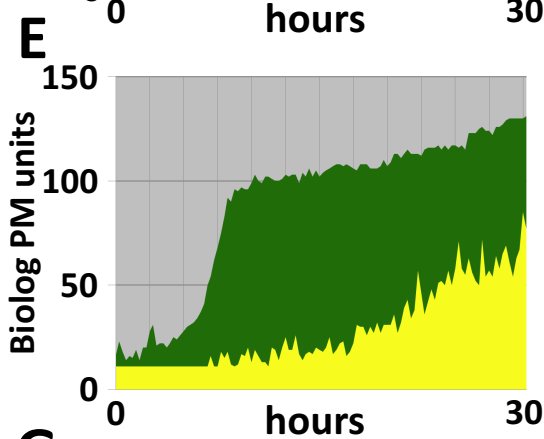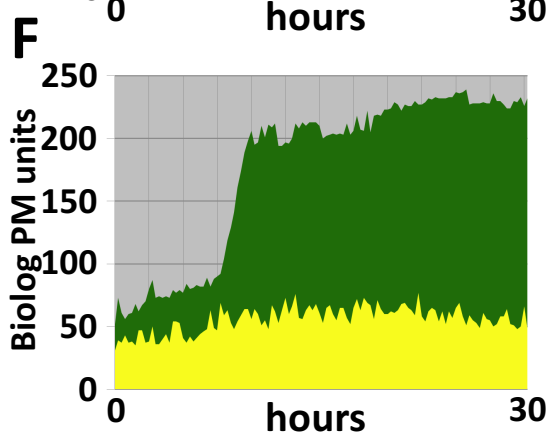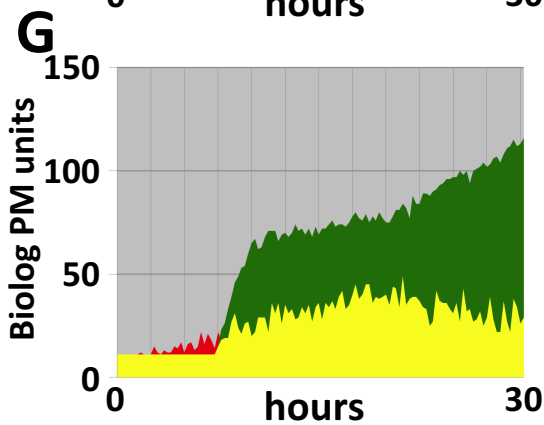

Supplement: mmc2 — Kinetic response curves parallelling bacterial growth for Biolog phenotype microarray antimicrobial tests in which the pTTQ18-Bcen2424_2356 plasmid facilitated a growth advantage. Curves for E. coli BL21 cells carrying pTTQ18 are shown in red, curves for BL21 cells carrying pTTQ18-Bcen2424_2356 are shown in green and regions of overlap in the response curves of these two strains are shown in yellow. The tests were conducted according to the manufacturer's instructions in the presence of 0.05 mM IPTG to promote expression of the cloned Bcen2424_2356 gene. The curves depict the colour intensity of a redox-active dye (y axis) over time (x axis; 30 h). (A) plate PM12, well E12 (benzethonium chloride) (B) plate PM14, well A3 (acriflavine), (C) plate PM14, well B3 (9-aminoacridine), (D) plate PM15, well E11 (methyl viologen), (E) plate PM18, well G7 (3,5-diamino-1,2,4-triazole [guanazole]), (F) plate PM18, well H12 (plumbagin), (G) plate PM19, well C4 (chlorhexidine). [file mmc2.pdf]
