# Supplementary material for: Pacing across the membrane: the novel PACE family of efflux pumps is widespread in Gram-negative pathogens
Source: Res Microbiol. 2018 Sep-Oct;169(7-8):450–4. doi: 10.1016/j.resmic.2018.01.001 (PMC6195760; doi:10.1016/j.resmic.2018.01.001)
Supplement: mmc3 — Putative substrates of PACE family proteins identified using whole cell transport experiments (chlorhexidine, acriflavine), conventional minimum inhibitory concentration analyses, (chlorhexidine, acriflavine, benzalkonium, dequalinium) and/or higher throughput resistance tests using the Biolog Phenotype Microarray system (chlorhexidine, acriflavine, benzethonium, 9-aminoacridine, methyl viologen, guanazole, plumbagin, domiphen). The chemical structures were obtained from the NCBI PubChem database and viewed using the web-based MolView tool (molview.org). [file mmc3.pdf]

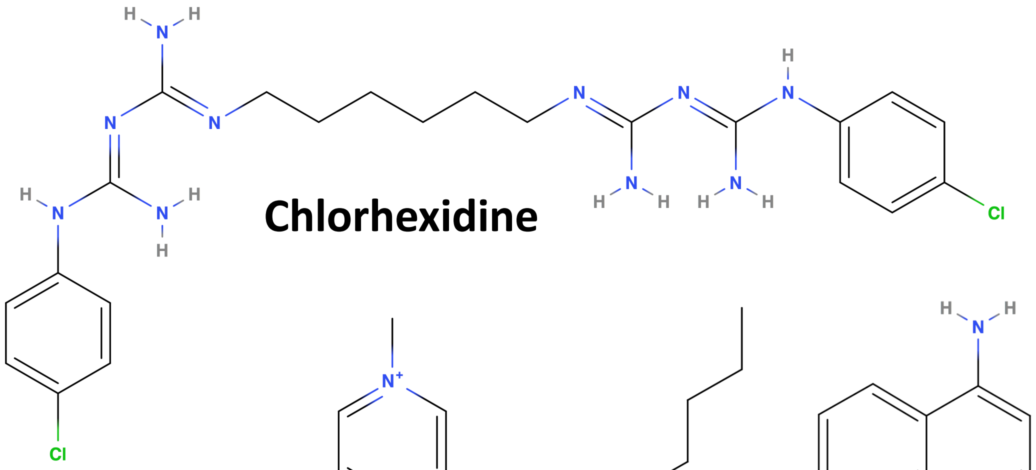

Chlorhexidine

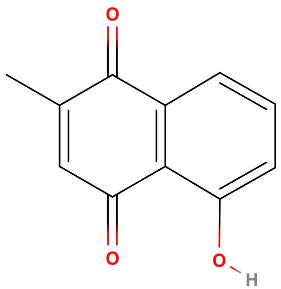

Plumbagin

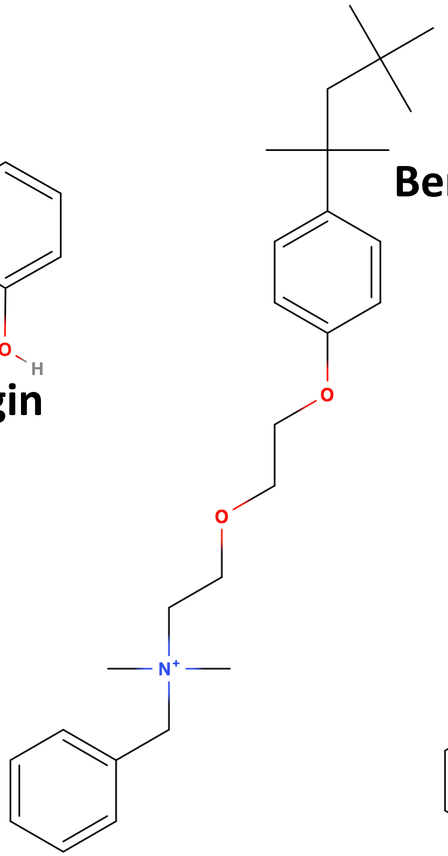

Benzethonium

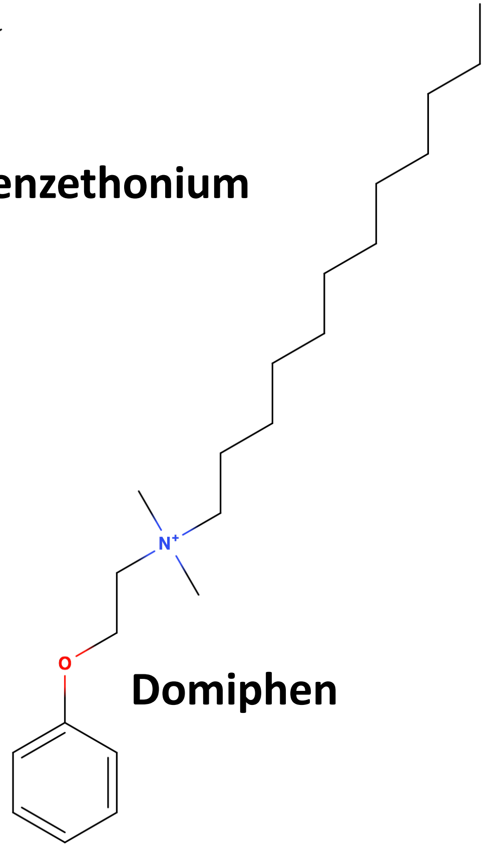

Domiphen

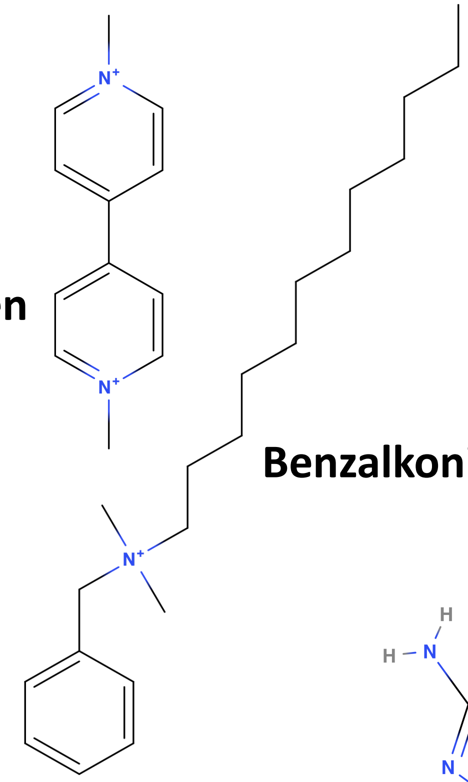

Methyl viologen

Benzalkonium

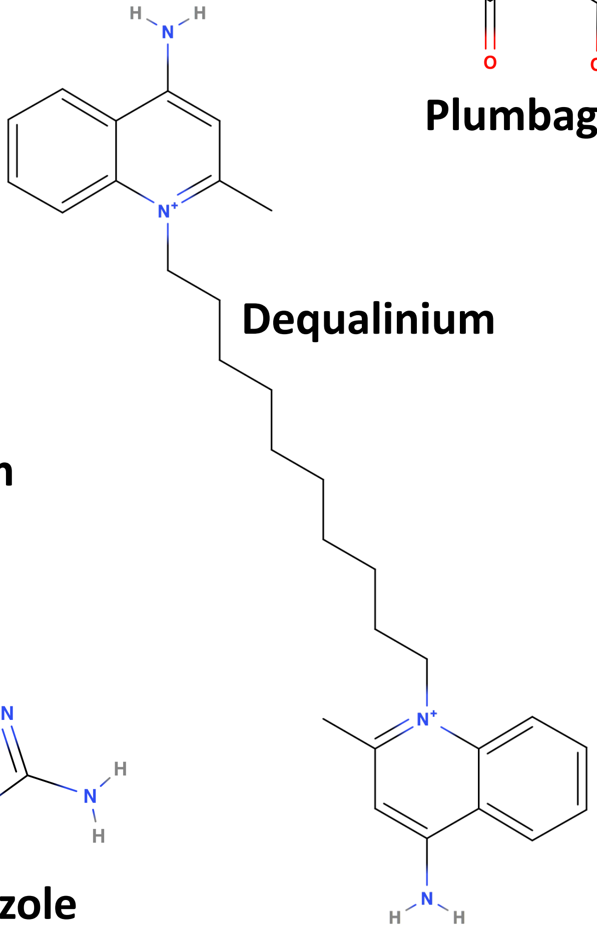

Dequalinium

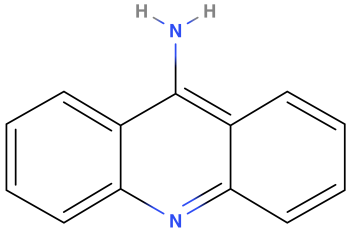

9-aminoacridine

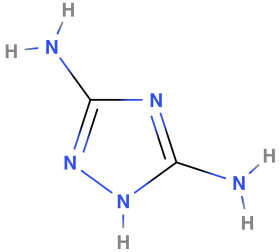

Guanazole

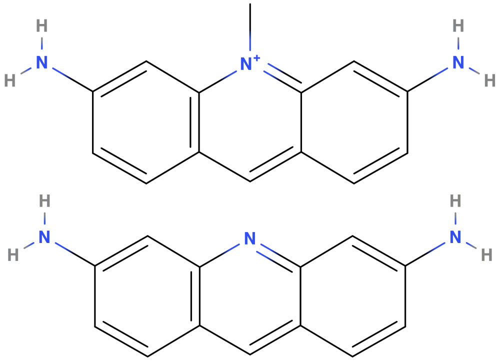

Acriflavine
